# Foreword to the special issue on the neuroscience of obesity and related disorders

**DOI:** 10.1007/s11154-022-09739-4

**Published:** 2022-06-13

**Authors:** Trevor Steward, Christina E Wierenga

**Affiliations:** 1 Melbourne School of Psychological Sciences, Faculty of Medicine, Dentistry and Health Sciences, University of Melbourne, Parkville, Victoria Australia; 2 Melbourne Neuropsychiatry Centre, Department of Psychiatry, The University of Melbourne, Victoria, Australia; 3 Department of Psychiatry, University of California, San Diego, San Diego, California USA; 4 Melbourne School of Psychological Sciences, Faculty of Medicine, Dentistry and Health Sciences, University of Melbourne, Redmond Barry Building #505, Parkville, Victoria Australia

## Introduction

Obesity represents one of the most critical public health crises facing the world today. Excess weight is associated with reduced quality of life and poorer mental health outcomes, as well as increased incidence of medical conditions such as diabetes, heart disease, stroke, and multiple types of cancer. Taking into consideration that most individuals with obesity who attempt to lose weight by restricting their caloric intake will not be successful, greater focus has been placed on examining the neurobiological drivers of obesity [1, 2]. In this Special Issue, we aim to provide an overview of neuroscience research that has been conducted on obesity. This collection highlights relevant new areas of exploration but is unable to cover every avenue of obesity-related neuroscience research due to the ever-expanding breadth of the field. It is our hope that this Special Issue can act as a useful resource for researchers and clinicians alike seeking to gain insights into the neurobiological factors underlying obesity. Similarly, we aim to spur discussions surrounding unresolved questions on how brain function contributes to obesity and to push the field forward by advocating for higher methodological standards in study design.

Obesity cannot be fully grasped without taking into account how an organism interacts with its food environment. Contreras-Rodriguez et al. [3] deliver a thorough discussion on how the remarkable growth in the consumption of ultra-processed foods and drinks (UPF) may be linked to the adverse health outcomes associated with obesity, including gut dysbiosis and metabolic dysfunction in regulating glucose. Their review covers evidence detailing how UPFs may modulate activity in the brain networks mediating eating behaviors and how the characteristics of UPFs - low nutrient but high energy density, and a high concentration of saturated fats, trans fats, and sugars - could hijack brain systems intended to limit overconsumption. Davidson and Stevenson [4] delve deeper into the impact of diet on brain function by describing a theoretical model linking Western-style diets to interference with a brain substrate that is central to processing of interoceptive signals of hunger and satiety. Their review illustrates findings from rats and humans demonstrating that the capacity of these signals to modulate the strength of appetitive and eating behavior depends on the functional integrity of the hippocampus and its learning and memory operations. Their framework argues that satiety provides an interoceptive context to inform organisms on whether or not food cues and appetitive behavior will be followed by rewarding outcomes after food intake, and that Western-style diets are associated with the emergence of pathophysiologies, which impair hippocampal-dependent learning and memory, thereby weakening food impulsive control.

Further support for the damaging impact of obesity on brain structure is provided in the review by Garcia-Garcia et al. [5]. In their review, the authors showcase how individuals with obesity demonstrate diminished grey matter volume and thickness most prominently in fronto-temporal regions of the brain. Concurrently, obesity is associated with reduced microstructural white matter integrity and increases in white matter hyperintensity load. The case is made for further research exploring how these structural alterations are attributable to the cardiometabolic complications that often coexist with obesity, such as low-grade systemic inflammation, hypertension, insulin resistance, or dyslipidemia. In terms of brain function, Parsons et al. [6] conducted a systematic review on functional magnetic resonance imaging studies (fMRI) examining resting-state functional connectivity in individuals with obesity. The authors framed their findings within a triadic model of problematic eating, centered on disrupted communication between reward, inhibitory, and homeostatic systems. They identified a pattern of increased orbitofrontal cortex and decreased insula cortex resting-state functional connectivity in individuals with obesity in comparison to healthy weight controls. Their systematic review concludes by offering methodological considerations to achieve more reliable resting-state functional connectivity findings.

Kung et al. [7] seek to expand neurobiological models of overeating by drawing on animal and human research to highlight how neural signaling encoding energy regulation, reward-learning, and habit formation are mediated by subcortical hypothalamic, brainstem, thalamic, and striatal regions. Emphasis is given to how ultra-high field 7-Tesla (7T) fMRI may be leveraged to shed light on the functional alterations in these subcortical systems and suggestions on how to investigate interactions of these systems with endocannabinoids and neuropeptides are offered. The utility of neuroimaging is taken a step further in Kozarzewski’s work advocating for the combined use of neuroimaging data with computational approaches to predict treatment response in obesity [8]. The authors argue that such an approach could deliver a prognostic tool to help predict the effectiveness of individual treatment methods so as to improve personalized medicine for patients seeking treatment. The case is made that additional studies comprising larger sample sizes and rigorous validation processes are first required to determine if these tools could be sufficiently accurate for clinical application. This work complements the review by Ester and Kullmann [9] summarizing the current state of human studies using transcranial direct current stimulation (tDCS), a non-invasive neurostimulation tool, to enhance self-control and influence food intake. Excitatory stimulation of the right dorsolateral prefrontal cortex (dlPFC) was found to be the most encouraging site in reducing food cravings to highly palatable food, whereas other studies report that stimulating the left dlPFC had effects on weight loss and weight maintenance, especially in multi-session approaches. The heterogeneity of these findings points to large interindividual differences in tDCS responsiveness and for the need for studies with larger samples to pinpoint individualized targets for non-invasive brain stimulation.

Godet et al.’s [10] review focuses on the brain correlates between emotions and eating behavior that may underpin emotional eating. The authors posit that prefrontal regions, the insula and reward pathways play a consequential role in both the cognitive control of emotions and in regulating subsequent eating behavior. In contrast, Kanoski and Boutelle [11] review evidence from both experimental rodent models and human studies to describe the behavioral and biological processes through which food-associated stimuli contribute to overeating and weight gain. By detailing findings from cue-potentiated feeding and Pavlovian-instrumental transfer models, they shed light on the neural circuits and peptide systems underlying food cue responsivity. Last, Guerrero-Hreins et al. [12] details the impact of bariatric surgery on disordered eating behaviors and synthesizes evidence suggesting that gut-derived signals, such as appetitive hormones, bile acid profiles, microbiota concentrations and associated neuromodulatory metabolites, influence neural circuitry implicated in food intake. This review underscores the clinical relevance of why understanding how changes in the gut-brain axis contribute to disordered eating incidence and severity after bariatric surgery is required in order to improve treatment outcomes.

To conclude, this special issue aims to serve as a helpful resource by providing updated reports on the state of neuroscience research in obesity and offering suggestions for improved methodological approaches to further advance scientific inquiry and mechanistic understanding of obesity.
